# Patterns of prescribing radiotherapy and bevacizumab in nationwide practice – analysis of 101 designated cancer care hospitals in Japan

**DOI:** 10.1093/jrr/rrv080

**Published:** 2015-12-09

**Authors:** Yoichiro Tsukada, Fumiaki Nakamura, Momoko Iwamoto, Atsuro Terahara, Takahiro Higashi

**Affiliations:** 1 Division of Health Services Research, Center for Cancer Control and Information Services, National Cancer Center, 5-1-1 Tsukiji, Chuo-ku, Tokyo 104–0045, Japan; 2 Department of Radiology, Toho University Omori Medical Center, Tokyo, Japan; 3 Department of Public Health/Health Policy, Graduate School of Medicine, the University of Tokyo, Tokyo, Japan

**Keywords:** adverse effects, bevacizumab, radiation-sensitizing agents, radiotherapy, Vascular Endothelial Growth Factor A

## Abstract

Radiotherapy and bevacizumab are each effective in treating patients with advanced cancer, but their concurrent use may cause serious adverse events (SAEs). Whereas sequential administration can theoretically reduce the risk of SAEs while maintaining the anticancer effects, this hypothesis remains unconfirmed, leading to variations in practice. To elucidate current practices, the patterns of care received by patients in Japan with regard to these two therapies were assessed in a large database of a hospital-based cancer registry linked with insurance claims. This database contained information on 106 057 patients diagnosed with seven major cancers in 2011 and the care they received up to the end of 2012. In total, 335 patients from 101 hospitals in the database were treated with both radiotherapy and bevacizumab. Of these patients, 50.8% had lung cancer, and 51.3% had Stage IV cancer. Of the 335 patients, 75 (22.4%) received these therapies concurrently. In patients treated sequentially, the time from the last dose of bevacizumab to the start of radiotherapy was most frequently 4–5 weeks (12.4%), whereas the time from the end of radiotherapy to the start of bevacizumab was most frequently 1–2 weeks (10.6%). The cumulative proportions of patients in these two groups receiving sequential therapies within 3 weeks were 19.0% and 26.1%, respectively. Many practices appeared to avoid the concurrent use of bevacizumab and radiation, but some provided concurrent therapy. Additional data are required to determine whether the avoidance of concurrent use should become a standard of care.

## INTRODUCTION

Neoplastic angiogenesis is essential for tumor growth [1]. Vascular endothelial growth factor (VEGF) is a heparin-binding growth factor expressed in tumor cells and adjacent endothelial cells of blood vessels, and is a key for inducing angiogenesis [1, 2]. Bevacizumab (Avastin®; Genentech, Inc.; South San Francisco, CA), an anti-VEGF monoclonal antibody approved for use by the US Food and Drug Administration (FDA) in 2004, inhibits tumor angiogenesis and the growth and proliferation of human tumor cell lines [3, 4]. Although bevacizumab alone cannot permanently control tumor growth in most patients [5], combinations of bevacizumab with systemic chemotherapy have improved outcomes in patients with a wide range of advanced or metastatic cancers, including colorectal cancer (CRC) [6], non–small-cell lung cancer (NSCLC) [7, 8], breast cancer [9] and renal cancer [10]. In Japan, bevacizumab was approved in July 2015 for patients with inoperable advanced or recurrent CRC, non-squamous NSCLC, breast cancer, ovarian cancer, and malignant glioblastoma. Moreover, clinical trials have assessed combinations of bevacizumab with radiotherapy in patients with several types of cancer, including rectal cancer [11], breast cancer [12], NSCLC [13], cervical cancer [14], and brain tumors [15, 16].

Bevacizumab, however, has been associated with several complications, such as intestinal perforation and delayed wound healing [17]. Because bevacizumab may delay wound healing, the FDA has recommended that bevacizumab be discontinued at least 28 days prior to elective surgery, and that this agent should not be restarted within 28 days after surgery and until the surgical wound has completely healed [18]. Furthermore, several clinical trials have reported that concurrent use of radiation therapy and bevacizumab may be associated with serious adverse events (SAEs) [19–22]. The FDA issued a Drug Warning against combined use in 2007 [19]. In real-world practice settings, however, conditions may arise necessitating simultaneous radiotherapy and bevacizumab. For example, CRC patients with liver metastases being treated with systemic chemotherapy and bevacizumab may experience severe abdominal pain arising from the tumor mass, and may therefore be considered for radiotherapy of the liver to control these symptoms [23]. At present, there are no professional guidelines for the use of simultaneous or sequential radiotherapy and bevacizumab, including the interval between the two treatments.

As a first step to understanding the type and frequency of SAEs associated with radiotherapy and bevacizumab, we analyzed the percentages and characteristics of cancer patients in Japan who were treated with both radiotherapy and bevacizumab, both simultaneously and sequentially, using a nationwide database.

## MATERIALS AND METHODS

### Data source

The database used was one that compiled health claims data linked to the Hospital-Based Cancer Registry (HBCR) from designated cancer care hospitals (DCCHs) and several hospitals that were not designated, but played a similar role in their communities and voluntarily submitted their data. This database was developed to measure process-of-care quality indicators for gastric, colorectal, breast, lung, liver, cervical and prostate cancer. In total, 178 hospitals joined the project: 173 of the 397 DCCHs and 5 other hospitals in 2011, and information on 106 057 patients was collected.

The insurance claims data were derived from an ongoing evaluation required of the hospitals that participate in a per-diem payment system (PDPS) based on diagnosis procedure combination (DPC) groups (DPC/PDPS) in the national health insurance scheme. The DPC/PDPS program was launched in 2002 and was adopted by secondary and tertiary care hospitals in Japan [24]. It includes unique identification codes for hospitals and patients, diagnoses, and the codes for all procedures and prescriptions supplied to each patient, as well as their dates. Although DPC/PDPS payments apply only to inpatient care, the evaluation collected the same information on outpatient care. This study included both inpatient and outpatient data.

The HBCR data contained information on cancer patients newly diagnosed, treated and followed up at the participating hospitals. The DCCHs, which are designated by the Ministry of Health, Labour and Welfare to provide specialized cancer care across communities in Japan, are required to submit their data annually to the National Cancer Center [25]. The entire HBCR database covered ∼70% of all newly diagnosed cancer patients in 2011 [25]. The data contain information on cancer patients and their clinical profiles, including clinical and pathological tumor-node-metastasis (TNM) stages, topology (site), histology codes of the International Classification of Diseases for Oncology, third edition (ICD-O-3), diagnosis and first-course treatment with dates of care.

The database collected insurance claims for at least one year, from each patient's first visit in 2011 to the hospital registered in the HBCR database, through December 2012. This study was approved by the Institutional Review Board of the National Cancer Center.

### Targeted patients

The database was searched for patients who received both external-beam radiotherapy (including intensity-modulated radiotherapy) and bevacizumab during the study period. Patients who received only stereotactic body radiotherapy (SBRT) were excluded, because the fees for SBRT were lump-sum payments and the dates of SBRT were unclear on some insurance claims data.

### Endpoints

Patients who received both radiotherapy and bevacizumab concurrently or sequentially were identified and classified into three groups: (i) a concurrent treatment (C) group, in which patients received concurrent radiotherapy and bevacizumab; (ii) a sequential bevacizumab–radiotherapy (B–R) group, in which the first dose of radiotherapy was delivered after the last dose of bevacizumab; and (iii) a sequential radiotherapy–bevacizumab (R–B) group, in which the first dose of bevacizumab was administered after the last irradiation. The characteristics of the three groups were compared, including cancer site, stage, and number of radiation fractions. Patients were subdivided into those who received <21 radiation fractions, generally considered palliative therapy, and ≥ 21 radiation fractions, generally considered therapy with curative intent. The cut-off of 21 was set based on the results of previous studies investigating palliative radiotherapy regimens [23, 26–29]. In the two sequential treatment groups, the intervals in weeks from the last day of radiotherapy to the first day of bevacizumab and from the last day of bevacizumab to the first day of radiotherapy were determined.

Intermittent irradiations at intervals of ≤30 days apart were categorized as belonging to a single course of radiotherapy, whereas intervals between two irradiations of >30 days were regarded as belonging to different courses of radiotherapy. If a patient underwent two or more courses of radiotherapy, the course nearest in time to bevacizumab treatment was considered. Stages were recorded according to the Union for International Cancer Control (UICC) TNM codes (UICC TNM classification of malignant tumors, 6th edition). Pathological stage was normally defined as each patient's final stage, although clinical stage was used when pathological stage was not known. Stata® version 13.1 software (StataCorp, College Station, TX) was used for all data analyses.

## RESULTS

Of the 106 057 cancer at 178 hospitals, 20 703 received radiotherapy, and 2447 received bevacizumab, with 335 patients at 101 hospitals receiving both. Table 1 shows the characteristics of these 335 patients. Of these, 170 (50.8%) had lung cancer, 172 (51.3%) had Stage IV disease, and 38 (11.3%) were >75 years old. A total of 75 patients (22.4%) were treated concurrently, including two who received two courses of concurrent treatment, and 260 (77.6%) were treated sequentially, including 137 who received bevacizumab followed by radiotherapy (B–R group), 142 who received radiotherapy followed by bevacizumab (R–B group), and 19 who received bevacizumab both before and after radiotherapy. The 335 patients received 356 courses of radiotherapy. Table 2 shows details of tumor sites, stages, and radiotherapy fractions per course. In all three groups, the most frequent subgroup consisted of patients with Stage IV lung cancer who were irradiated with <21 fractions.

**Table 1.** Characteristics of patients treated with radiotherapy and bevacizumab (*n* = 335)

Patient characteristics	
Number of patients, *n* (%)	
Treated concurrently	75 (22.4)
Treated sequentially	260 (78.6)
Mean (±standard deviation) age, years	62.2 (±11.1)
<75 years, *n* (%)	297 (88.7)
≥75 years, *n* (%)	38 (11.3)
Sex: female, *n*, %	106 (31.6)
Sites of cancer, %	
lung	171 (51.0)
rectal	89 (26.6)
colon	37 (11.0)
breast	28 (8.4)
others	10 (3.0)
UICC stage, *n,* %	1 (0.3)
0	1 (0.3)
I	18 (5.4)
II	27 (8.1)
III	102 (30.5)
IV	172 (51.3)
unknown	15 (4.5)

**Table 2.** Number of radiotherapy courses in each group by cancer type, stage, and irradiation fractions

	Stage 0		Stage I		Stage II		Stage III		Stage IV		Unknown	
	<21^a^	≥ 21^b^	<21^a^	≥ 21^b^	<21^a^	≥ 21^b^	<21^a^	≥ 21^b^	<21^a^	≥ 21^b^	<21^a^	≥ 21^b^
**Concurrent treatment group^c^**												
Gastric cancer, *n*	0	0	0	0	0	0	0	0	0	0	1	0
Colon cancer, *n*	0	0	0	0	0	1	1	2	3	0	1	0
Rectal cancer, *n*	0	0	0	2	1	5	1	16	6	4	0	1
Lung cancer, *n*	0	0	0	1	0	0	0	1	25	3	0	0
Breast cancer, *n*	0	0	0	0	0	0	0	1	0	1	0	0
**B­R group^d^**												
Gastric cancer, *n*	0	0	1	0	0	0	0	0	0	0	0	0
Colon cancer, *n*	0	0	0	0	0	0	1	0	16	0	2	0
Rectal cancer, *n*	0	0	0	0	0	1	2	3	11	4	3	0
Liver cancer, *n*	0	0	0	0	0	0	0	1	0	0	0	0
Lung cancer, *n*	0	0	1	2	1	1	18	6	46	4	0	0
Breast cancer, *n*	0	0	1	0	0	0	2	5	1	1	0	0
Cervical cancer, *n*	0	0	1	0	0	0	0	0	0	0	0	0
Prostate cancer, *n*	0	0	0	0	0	2	0	0	0	0	0	0
**R–B group^e^**												
Gastric cancer, *n*	0	0	1	1	0	0	0	0	0	0	0	0
Colon cancer, *n*	0	0	0	0	1	1	1	1	4	0	3	2
Rectal cancer, *n*	0	0	1	1	0	2	1	11	9	5	0	1
Lung cancer, *n*	0	0	3	3	1	4	6	19	34	7	0	0
Breast cancer, *n*	0	0	0	0	5	1	2	4	2	1	1	1
Cervical cancer, *n*	1	0	0	0	0	0	0	0	0	0	0	0
Prostate cancer, *n*	0	0	0	0	1	0	0	0	0	0	0	0

^a^<21 fractions of radiotherapy, ^b^ ≥ 21 fractions of radiotherapy, **^c^**Patients receiving concurrent radiotherapy and bevacizumab **^d^**Patients receiving radiotherapy after the last dose of bevacizumab, **^e^**Patients receiving bevacizumab after the last irradiation.

### Intervals from bevacizumab to radiotherapy

**Fig. 1.**
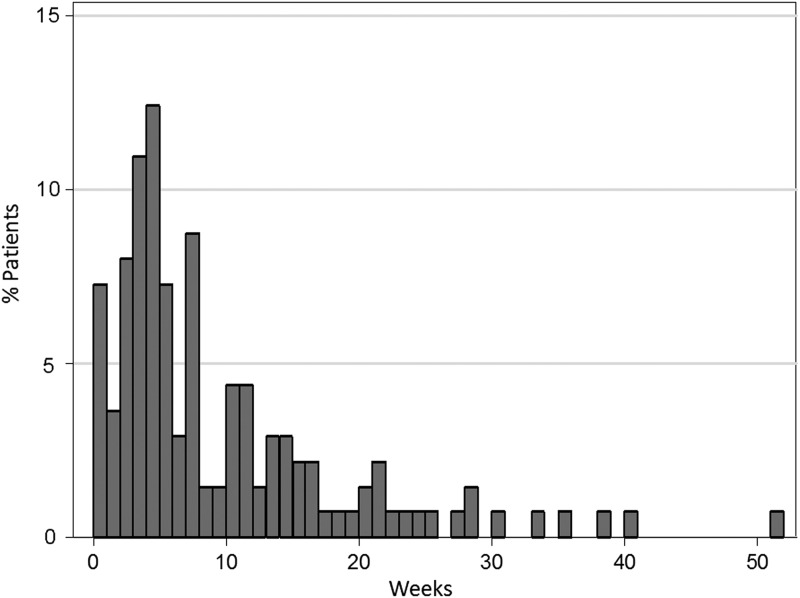
Percentage of patients in the B–R group subdivided by time intervals from the last dose of bevacizumab to the first dose of radiotherapy.

Figure 1 shows the proportion of patients in the B–R group by weekly intervals from the last administration of bevacizumab to the first dose of radiotherapy. The most common interval was 4–5 weeks (17 patients, 12.4%), although 26 patients (19.0%) started radiotherapy within 3 weeks after the last dose of bevacizumab.

### Intervals from radiotherapy to bevacizumab

**Fig. 2.**
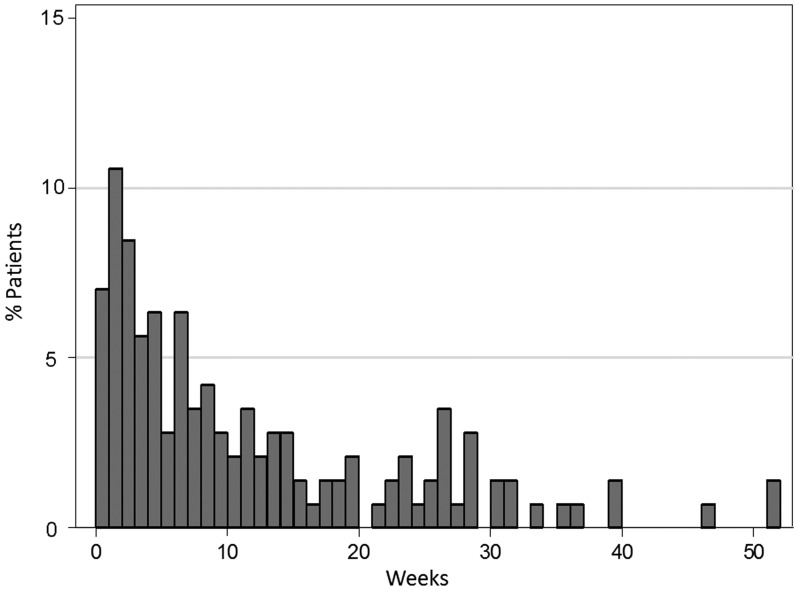
Percentage of patients in the R–B group subdivided by time intervals from the last dose of radiotherapy to the first dose of bevacizumab.

Figure 2 shows the proportion of patients in the R–B group by weekly intervals from the last dose of radiotherapy to the first dose of bevacizumab. The most common interval was 1–2 weeks (15 patients, 10.6%), with 37 patients (26.1%) starting bevacizumab within 3 weeks after the last dose of radiotherapy.

## DISCUSSION

This study showed that about only one-fifth of the cancer patients in Japan who were treated with both radiotherapy and bevacizumab received the two concurrently. In most patients, the interval between finishing one treatment and starting the other was several weeks, indicating that many practices throughout Japan avoided concurrent treatment with radiotherapy and bevacizumab treatment. To our knowledge, this is the first study to assess patterns of radiotherapy and bevacizumab treatment in cancer patients. These patterns may be associated with the occurrence and numbers of SAEs, as well as with factors affecting the occurrence of SAEs.

Several clinical trials have reported SAEs in patients concurrently treated with radiotherapy and bevacizumab [20–22]. For example, one study analyzed SAEs in two independent Phase II clinical trials in patients with limited small-cell lung cancer and locally advanced NSCLC [20]. In the first trial, in which patients received chemoradiation therapy with concurrent bevacizumab, two of 29 patients experienced tracheoesophageal fistula (TEF) formation, one resulting in death, and another patient died from an aerodigestive hemorrhage. In the second trial, two of five patients experienced TEF formation after chemoradiation therapy with concurrent bevacizumab. In a Phase I trial, four of six patients with inoperable Stage III NSCLC who received induction chemotherapy followed by concurrent thoracic radiotherapy with bevacizumab developed pulmonary toxicities [21]. All three trials were terminated early due to these SAEs [20, 21].

Recent *in vitro* studies reported that bevacizumab can sensitize cancer cells to radiotherapy, resulting in downregulation of VEGF expression, inability to repair double-strand breaks in DNA, and normalization of tumor microvessels, resulting in tumoricidal effects [30, 31]. The combination of anti-VEGF antibody, irradiation, and bleomycine in mouse models resulted in enhanced intestinal injury with severe epithelial ulcerations during the acute phase, and lung fibrosis during the late phase [32]. Anti-VEGF antibody inhibits VEGF signaling pathways required for wound-healing processes following normal tissue damage by radiation, suggesting caution in treating patients with combinations of targeted agents and radiotherapy.

Our findings showed that intervals of several weeks elapsed between bevacizumab and radiation treatment in many sequentially treated patients. These intervals tended to be longer in the B–R than in the R–B group, perhaps to avoid the adverse events associated with any remaining bevacizumab. However, about one-fifth of patients in the B–R group started radiotherapy within 3 weeks, the estimated half-life time of bevacizumab in the human body [18]. Moreover, about one-fourth of the patients in the R–B group started bevacizumab within 3 weeks after finishing radiotherapy. A Phase III trial that showed that the addition of improved overall survival in patients with advanced cervical cancer and a history of cisplatin-based chemoradiation therapy utilized a wash-out period of at least 3 weeks from the last dose of radiotherapy to remove potential deleterious effects of these previous treatments [33]. Furthermore, more than 10% of our patients were ≥ 75 years old, and large numbers of patients in all three groups had Stage IV lung cancer and were irradiated with fewer than 21 fractions. These characteristics suggest that this treatment was mainly used for palliative purposes, including symptom management, in patients with advanced cancers, and that these patients were at higher risk of SAEs than patients in better overall condition who received regular therapy. Further studies, analyzing SAEs and their risk factors, are required to determine the standards of care in these patients, balancing the risks and benefits of treatment.

Our study had several limitations. First, our analyses used an insurance claims database. The claims data did not include the site or the total dose of irradiation, suggesting the need for further studies to assess these factors. Second, we classified radiotherapy courses into two groups, with a cut-off of 21 fractions. Although some patients may have been misclassified, we believe this analysis is useful in gaining insight into the purposes of radiotherapy. Third, our analysis included data from patients with seven major types of cancer treated at 178 hospitals, including 173 DCCHs, suggesting that these results may not have been representative of cancer patients throughout Japan. However, the database included 106 057 patients, about one-fifth of those newly diagnosed in 2011 with these seven major types of cancer in Japan [34], suggesting that our findings represent a trend of care practices in Japan. Finally, we analyzed patients with seven major types of cancer, suggesting that these findings may not be applicable to patients with other types of cancer, such as ovarian cancer and brain tumors.

## CONCLUSION

In conclusion, many oncology practices in Japan appeared to avoid concurrent treatment with bevacizumab and radiotherapy. Further studies are needed to determine whether the avoidance of concurrent use should become the standard of care.

## FUNDING

This work was supported by the National Cancer Center Research and Development Programs (Grant number H25-A-21). Funding to pay Open Access publication charges for this article was also provided by this grant.
